# Mechanisms of Resistance to Endocrine Therapy in Breast Cancer: Focus on Signaling Pathways, miRNAs and Genetically Based Resistance

**DOI:** 10.3390/ijms14010108

**Published:** 2012-12-20

**Authors:** Rocío García-Becerra, Nancy Santos, Lorenza Díaz, Javier Camacho

**Affiliations:** 1 Department of Reproductive Biology, National Institute of Medical Sciences and Nutrition Salvador Zubirán, Vasco de Quiroga No. 15, Tlalpan, México 14000, D.F., Mexico; E-Mails: rocio.garciab@quetzal.innsz.mx (R.G.-B.); santos_2105@hotmail.com (N.S.); lorenzadiaz@gmail.com (L.D.); 2 Department of Pharmacology, Center for Research and Advanced Studies, Avenida Instituto Politécnico Nacional 2508, México 07360, D.F., Mexico

**Keywords:** endocrine therapy, breast cancer, estrogen receptor, hormonal resistance

## Abstract

Breast cancer is the most frequent malignancy diagnosed in women. Approximately 70% of breast tumors express the estrogen receptor (ER). Tamoxifen and aromatase inhibitors (AIs) are the most common and effective therapies for patients with ERα-positive breast cancer. Alone or combined with chemotherapy, tamoxifen significantly reduces disease progression and is associated with more favorable impact on survival in patients. Unfortunately, endocrine resistance occurs, either *de novo* or acquired during the course of the treatment. The mechanisms that contribute to hormonal resistance include loss or modification in the ERα expression, regulation of signal transduction pathways, altered expression of specific microRNAs, balance of co-regulatory proteins, and genetic polymorphisms involved in tamoxifen metabolic activity. Because of the clinical consequences of endocrine resistance, new treatment strategies are arising to make the cells sensitive to tamoxifen. Here, we will review the current knowledge on mechanisms of endocrine resistance in breast cancer cells. In addition, we will discuss novel therapeutic strategies to overcome such resistance. Undoubtedly, circumventing endocrine resistance should help to improve therapy for the benefit of breast cancer patients.

## 1. Introduction

Estrogens play an important role in the pathogenesis of breast cancer through the estrogen receptor alpha (ERα). Approximately 70% of breast tumors are ER-positive (+) [1]. Consequently, therapies aim to either reduce estrogen levels or to block signaling through ERα. Three classes of antihormone endocrine agents have been used: selective estrogen receptor modulators (SERMs, e.g., tamoxifen) that block the activity ER; estrogen synthesis inhibitors (e.g., aromatase inhibitors (AIs) such as anastrozole, letrozole, and exemestane); and selective estrogen receptor downregulators (SERD, e.g., ICI 182,780 (Fulvestrant) and ICI 164,384) that induce destabilization and degradation of ER. These approaches have proven clinical efficacy in women with ER(+) breast cancer [2].

For more than three decades, tamoxifen has been the basis of hormonal therapy in both early and advanced breast cancer patients [3]. However, almost 50% of patients with advanced disease do not respond to first line treatment with tamoxifen (*de novo* resistance); furthermore, a significant percentage of patients experience tamoxifen relapse, despite an initial positive drug response (acquired resistance) [4,5]. This differential response might reside in the expression (*de novo* or acquired) of specific molecules involved in different signaling pathways, which eventually could be used as predictive biomarkers of resistance. Moreover, these markers may be used to select patients that might benefit from additional targeted treatments aside from ER [6,7].

Tamoxifen resistance occurs in breast cancer patients and is the main problem limiting the efficacy of the treatment. AIs therapy (either as initial treatment or sequentially after tamoxifen) seems to produce more benefits than the use of tamoxifen alone and might be effective in tamoxifen-resistant patients. Nevertheless, the response rate to these compounds is only slightly higher as compared with tamoxifen in patients with advanced breast cancer, and both *de novo* and acquired resistance to AIs also occur [8–10]. Recently, fulvestrant has demonstrated clinical efficacy among patients who relapsed for a second time after responding to tamoxifen and AIs; more investigations are being conducted to explore the clinical potential of this approach [11,12].

However, despite the incorporation of more potent endocrine agents, resistance to all forms of endocrine therapy remains a major problem. A better understanding of the molecular mechanisms of endocrine resistance might enable the use of novel strategies for therapeutic intervention. The aim of this review is to summarize some of the key novel findings on the mechanisms of endocrine resistance and its therapeutic implications. First, we will provide a general overview of ERs. Then, we will focus on the different mechanisms proposed in hormonal resistance and discuss several examples of combined therapy as a potential approach to overwhelm such resistance. Finally, we will provide some conclusions and remarks on the strategies and potential future directions in this cancer field.

## 2. ER Action and Function

ER belongs to a superfamily of nuclear receptors that serve as transcription factors [13]. ERα and ERβ are produced by distinct genes located on chromosome 6 and 14, respectively [14–16] Both receptors are present in normal breast tissue, but only ERα is associated with breast cancer initiation and progression, while ERβ function in breast cancer is still unclear [17]. However, several studies have described that ERβ exerts an opposite effect to ERα, inhibiting the ability of estrogens to stimulate proliferation. Actually, ERβ impaired expression contributes to tumor progression [18]. Accordingly, high ERβ expression has been correlated with better survival [19].

**Figure 1:**
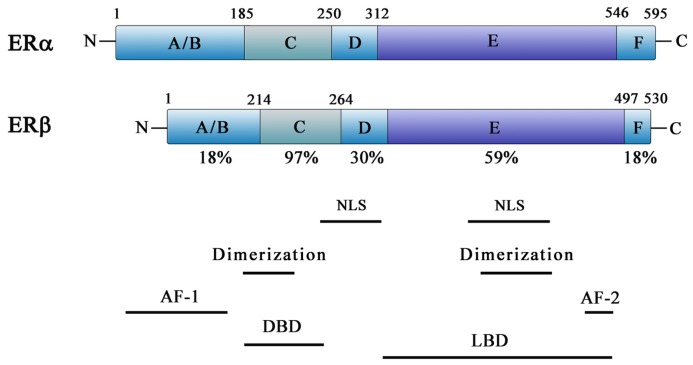
Schematic representation of functional domains of human ERα and ERβ. The A/B domain at the *N*-terminal contains AF-1 site. The C domain includes the DNA-binding domain (DBD) and a dimerization site. The D domain contains a nuclear localization signal. The E/F domain is located at the *C*-terminal and comprises the ligand binding, as well as the AF-2 domain, a second nuclear localization signal, and another dimerization site.

Both receptors share a common structural architecture; they are composed of six domains, designated A–F (Figure 1) [20,21]. The *N*-terminal or A/B domain contains the activation function 1 (AF1), a region responsible for the transcriptional activity of ER in the absence of ligand [17,22]. The C domain, known as the DNA-binding domain (DBD), is composed by a two zinc-finger structure that plays an important role in receptor dimerization and binding of receptors to specific DNA sequences [14,23–26]. The D domain is a hinge region between the C and E domains and includes a nuclear localization signal [27,28]. The E domain, referred to as ligand-binding domain (LBD), consists of a second nuclear localization signal, a dimerization site and a twelve-helix region involved in ligand binding [29]. The E domain also harbors the activation function 2 (AF2), responsible for the ligand-dependent activation of ER [30,31]. The F domain located at the *C*-terminal is a small region that modulates both AF1 and AF2, although it is unnecessary for transcriptional activation [17,22,31]. ERα and ERβ have a high degree of homology in their DBD (97% amino acid identity) and LBD (59% amino acid identity) [32,33].

ERα is associated to cell proliferation and survival through two different mechanisms: genomic and non-genomic signaling pathway. The classical genomic pathway is the most characterized ER signaling pathway and is initiated by the ligand binding to its receptor. The binding induces a conformational change and dissociation of their chaperon proteins, leading to receptor dimerization and translocation to the nucleus [17]. This ligand-ER complex binds to the consensus sequence, known as estrogen-response element (ERE), directly or indirectly through protein-protein interactions with activator protein 1 (AP1) or SP 1 sites in the promoter region of target genes, resulting in recruitment of coregulatory proteins (coactivators or corepressors), which can either enhance or repress ER transcriptional activity depending on the specificity of the ligand [34,35]. Microarray analysis showed that approximately 70% of estrogen-responsive genes were downregulated by estradiol in MCF-7 cells, those genes were transcriptional repressors or anti-proliferative and pro-apoptotic genes, while genes involved in the induction of cell proliferation were upregulated [36].

In addition to their classic genomic actions mediated through activation of nuclear ERs, estrogens can also elicit rapid responses that are often non-genomic. These responses are mediated via ERs localized either near or at the plasma membrane within caveolar rafts, as well as in other membrane domains [37–39]. The plasma membrane ER may interact with many other proteins including adaptor proteins, G-proteins, Src, growth factor receptors (EGFR, IGFR1, HER2), cytoplasmic kinases (MAPKs, PI3K, AKT), and signaling enzymes (adenyl cyclase, nitric-oxide synthase), mediating mechanisms independent of gene transcription [40–44]. Some of these non-genomic estrogen actions have been attributed to either ERs or its truncated forms. However, the orphan GPCR-like protein, GPR30 (G protein coupled receptor 30) [45,46], recently renamed GPER [47], has also been suggested to be involved. Several data support a role for GPER as an intermediary element in the rapid, non-genomic actions of estrogens [48].

Both genomic and non-genomic pathways interact with each other in crosstalk. Whereas nuclear ER induces the expression of transforming growth factor (TGF)α and amphiregulin (AR), both TGFα and AR are able to bind and stimulate EGFR or EGFR/HER2 and consequently activate MAPK and AKT [34–36]. On the other hand, membrane ER can bind to caveolin 1 and activate specific G proteins and src, which activates matrix metalloproteinases that cleave transmembrane precursors of heparin binding-EGF (HB-EGF), an EGFR ligand [27]. In addition, several cytokines, growth factors, EGFR ligands, IGF1R and pathways such as MAPK/ERK, PI3K/AKT, p90rsk and p38 MAPK phosphorylate ER at key positions in the AF-1 (serines 118 and 167, and threonine 311) and other domains; this process is defined as ligand-independent activation and results in the activation of the ER genomic pathway [49–53]. It has been demonstrated that estrogen acts through human GPER to activate a stimulatory G protein, resulting in the stimulation of adenylyl cyclase activity and increased cAMP production by plasma membranes of ER-negative (−) cells and GPER-transfected cells. In turn, the estrogen/GPER complex can also release epidermal growth factor, EGF-related ligands, and transactivate the EGF receptor (EGFR) [54,55]. To date, GPER has been implicated in the development and progression of breast cancer [56,57]. Thus, it is important to consider both genomic and non-genomic signaling pathways of ER in the regulation of intracellular signaling cascades that ultimately contribute to breast cancer progression.

## 3. Mechanisms of Endocrine Resistance

Several mechanisms of resistance to endocrine therapy have been hypothesized and a include loss of ERs, different signaling pathways, altered expression of microRNAs, as well as tamoxifen metabolism. In addition, both genomic and non-genomic crosstalk and the complex interrelation between the estrogen receptors subtypes and growth factors contribute to endocrine resistance developing [58]. In the following, we will describe the main proposed mechanisms responsible for endocrine resistance.

### 3.1. Loss or Modification in the ER Expression

ER expression is the main predictor of response to endocrine therapy, and lack of expression of ER is the principal mechanism of *de novo* resistance to hormonal therapy. Several mechanisms have been proposed to explain the absence of ER expression. These mechanisms involve epigenetic changes such as aberrant methylation CpG islands of the ER promoter and histone deacetylation, resulting in a compact nucleosome structure that limits transcription [59–62]. The co-treatment with inhibitors of DNA methyltransferase-1 (DNMT-1, such as 5-aza-2-deoxycytidine (AZA)) and histone deacetylase (HDAC, such as Trichostatin A (TSA) and suberoylanilide hydroxamic acid (SAHA)) induce ER gene expression in ER(−) breast cancer cells and restore sensitivity to antiestrogen [59,63–66]. In ER(−) MDA-MB-231 cells, which overexpress EGFR, SAHA may not only reactivate silenced ER, but also simultaneously deplete EGFR expression and abolish EGF-initiated signaling pathways including phosphorylated PAK1, p38MAPK and AKT [67]. *In vitro* and *in vivo* studies showed that the treatment with the histone deacetylase inhibitor entinostat (ENT) increased the expression of ERα and aromatase. Notably, ERα and aromatase upregulation resulted in sensitization of breast cancer cells to estrogen and letrozole [68]. Moreover, Scriptaid (a novel HDAC inhibitor) has also shown to cause re-expression of functional ER, induce inhibition of tumor growth, and sensitize hormone-resistant breast cancer cells to tamoxifen [69,70].

It has been also hypothesized that loss of ER expression might be responsible for acquired resistance to endocrine therapy. However, only 17%–28% patients with acquired resistance do not express ER [71–73], and ER(+) patients responding initially to tamoxifen, usually do not lose expression of ER after developing resistance to therapy. Approximately 20% of patients respond to second-line treatment with either AIs or fulvestrant, which may mean that patients with acquired resistance to tamoxifen still express ER [74,75].

Other mechanisms proposed in the loss of ER expression are hypoxia, overexpression of EGFR or HER2, MAPKs hyperactivation, and involvement of p53 and pRb2/p130. Hypoxia induces proteasome-dependent degradation of ER in ZR-75 breast cancer cells, leading to decreased protein levels [76]. High expression of EGFR and HER2 in ER(−) breast cancers were found, suggesting that activation of growth factor signaling and consequently, the activation of MAPK might contribute to transcriptional repression of ER gene, resulting in endocrine resistance [77,78]. Recent results showed that p53 upregulates ER gene expression through elements located upstream of the ER promoter. Interestingly, a high percentage of breast tumors with p53 mutations are ER(−). These studies suggest that specific p53 mutations in breast tumors may contribute not only to oncogenesis but also to hormonal resistance. Furthermore, the treatment of MCF-7 cells with paclitaxel resulted in the induction of ER gene transcription, which may be mediated through the induction of p53 [79,80]. On the other hand, pRb2/p130 also plays an important role in the transcriptional regulation of the ER promoter by recruiting multi-molecular transcriptional complexes, and may be considered as a promising target in breast cancer treatment, especially for ER(−) tumors [81].

Modifications in ER expression such as mutations might affect the response to anti-estrogens. For example, substitution of aspartate by tyrosine at position 537 in the ligand-binding domain causes ER activation in the absence of the ligand [82]. The change of aspartate to tyrosine at amino acid 351 in the ER has been identified in a tamoxifen-stimulated cell line [83], and modification of lysine 303 to arginine results in increased ER sensitivity to estrogens [84]. This type of mutation might alter ER function, however, only 1% of primary breast cancer patients carry these mutations [85] and endocrine resistance also occurs even in the absence of these mutations.

Another mechanism that is associated with SERM resistance is the altered expression of ERβ. High levels of ERβ are found in pre-invasive mammary tumors of tamoxifen-resistant patients [86]. However, other studies have reported that ERβ has a negative effect on ERα promoted transcription and that low levels of ERβ may contribute to endocrine resistant [87,88]. Then, the role of ERβ in the resistance to therapy is still controversial.

### 3.2. Epigenetics Mechanisms Regulating ER Expression

Several epigenetic mechanisms that regulate the expression of many genes also regulate ER expression. Nowadays, epigenetic studies could provide new biomarkers to predict and diagnose acquired resistance in response to treatment [89].

Analysis of ERα methylation status offers an alternative to determine whether endocrine therapy will be effective in breast cancer patients. Loss of ERα expression is often associated with promoter hypermethylation of ERα gene [90,91]. Similarly, PR methylation has been observed in endocrine resistant tumors [92]. Several studies have reported that a high expression ratio of Homeobox protein (HOXB13)/Interleukin-17B receptor (IL17BR) predicts tumor recurrence in node-negative, ER(+) breast cancer patients treated with tamoxifen. While the details of deregulation of HOXB13 and IL17BR are still under investigation, there is evidence that expression of HOXB13 is under epigenetic control [93]. Patients with ER(+) breast tumors with low levels of CDK10 (Cyclin-dependent kinase 10) relapsed early under tamoxifen treatment. Importantly, this downregulated CDK10 expression is associated with methylation of the CDK10 promoter, suggesting CDK10 gene methylation as a novel marker for determining response to endocrine therapy [94,95]. At least three studies have used high-throughput DNA methylation profiling to associate DNA methylation of candidate genes with outcome of breast cancer patients after tamoxifen therapy [96].

Several inhibitors of enzymes controlling epigenetic modifications, specifically DNMT and HDACs, have been developed and shown promising anti-tumorigenic effects [89,91,96]. Epigenetic changes are frequent events, and unlike genetic mutations, epigenetic modifications are reversible events; thus, the inhibition of these mechanisms could be a potential therapeutic strategy for the treatment of endocrine resistant breast cancer, either through direct effects on epigenetic changes, or by modulating known targets of other therapies.

### 3.3. Regulation of Signal Transduction Pathways

**Figure 2:**
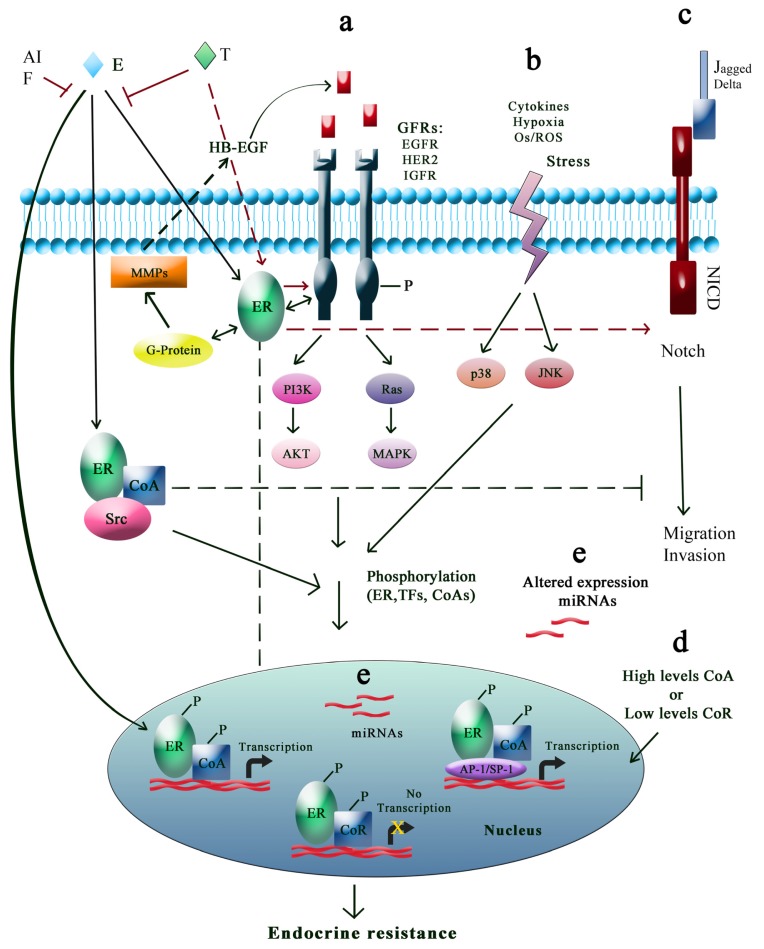
Pathways involved in endocrine resistance. (**a**) While tamoxifen (T), aromatase inhibitors (AIs), or fulvestrant (F) inhibit estrogen (E) signalization, GFR pathways promote ER phosphorylation, transcription factors (TFs), and their coactivators (CoA) in a ligand-independent manner. E-ER complex outside the nucleus can interact with GFRs, Src, CoA and matrix metalloproteinases that release heparin-binding-EGF; (**b**) Stress may trigger signalization leading to ER and its coregulators phosphorylation; (**c**) Notch regulates the migration and invasion of breast cancer cells. E inhibits this pathway while T activates it; (**d**) High levels of CoA, low levels of corepressors (CoR) and altered expression of miRs (**e**) have been implicated in endocrine resistance development.

Crosstalk between ER and different signaling pathways, such as growth factor receptor, cell survival (PI3K/AKT), stress and/or cytokine signaling pathway, have been implicated in acquired and intrinsic resistance to endocrine agents, and are represented in Figure 2.

#### 3.3.1. Growth Factor Receptor Signaling Pathways

The growth factor receptor signaling pathways can stimulate cancer growth either in concert with ER signaling or bypassing it. Preclinical and clinical evidence suggests that growth factor receptor signaling contributes to endocrine resistance. Both the ER and growth factor receptor pathway act in crosstalk, where the membrane ER can activate growth factor receptor signaling, in turn, this can phosphorylate ER and its coregulator proteins [97]. Phosphorylation of key serine residues within the activator function-1 (AF-1) domain of ER, in particular serine 118 and 167, can promote re-activation of ER function in a ligand-independent manner and contribute to transcription of estrogen-sensitive genes in the presence of antiestrogen agents [50,51,98]. Reporter gene construct studies in tamoxifen resistant cells indicate that the EGFR/MAPK-promoted ER AF-1 phosphorylation enhances the agonistic behavior of tamoxifen, resulting in the expression of estrogen regulated genes [99]. It has also been shown that growth factor signaling pathways promote phosphorylation of ER coactivators [100]. Indeed, overexpression of the co-activator AIB1 correlates with resistance to tamoxifen in breast cancer patients and in the EGFR/HER2/MAPK-dependent phosphorylation, and it has been proposed to mediate tamoxifen resistance in HER2 overexpressing MCF-7 cells [101].

Overexpression and activation of growth factor receptors, such as EGFR, HER2 and IGF1R, drive the proliferation and survival through activation of MAPK and PI3K/AKT signaling pathways in endocrine-resistant breast cancer [102]. One of the first indications of the crosstalk between ER and growth factors receptors was the transfection of ER(+) breast cancer cells with HER2, resulting in downregulation of ER and resistance to tamoxifen [103,104]. Subsequently, several works have supported a role for HER2 in intrinsic resistance to endocrine therapy. In MCF-7/HER2-18 cells manipulated to overexpress HER2 as well as ER, tamoxifen behaves as an estrogen agonist, stimulating their growth. Phosphorylation and activation of both ER and EGFR/HER2 receptors as well as MAPK and AKT signaling pathways, and recruitment of AIB1 coactivator were increased in these cells compared with wild-type MCF-7 cells, this observation was also supported in xenograft tumors [97]. The potential involvement of this crosstalk has been also observed in a meta-analysis of clinical trials in which patients with metastatic ER(+)/HER2(+) breast cancer were treated with tamoxifen after a shorter time of treatment failure in comparison to HER2(−) cancers [105]. In tumors that acquired resistance to fulvestrant, there was also a marked upregulation of HER-2 and the downstream signaling molecule MAPK, suggesting that this pathway may mediate the resistant phenotype [106]. Notably, breast cancer cells resistant to exemestane (AIs) induce amphiregulin expression in an ER-dependent manner. A possible mechanism of resistance to exemestane is that amphiregulin activates the EGFR pathway and leads to the activation of the MAPK pathway that drives cell proliferation [107]. Interestingly, in a xenograft model of ER(+)/HER2 overexpressing breast cancer, the loss of the ER protein and deregulation of the progesterone receptor with increased mucins, especially MUC4, which has been associated with endocrine resistance in breast cancer, has been observed [108].

Therefore, the use of either specific inhibitors or blockers of growth factor receptors has been one of the most promising therapeutic approaches in breast cancer. Gefitinib, a selective inhibitor of EGFR, restores the effects of tamoxifen in HER2-overexpressing tamoxifen-resistant MCF-7 cells, while trastuzumab, a monoclonal antibody that blocks HER2, can inhibit proliferation of endocrine resistant ZR-75-1 cells [97]. Furthermore, the HER2 tyrosine kinase inhibitor AG1478 and trastuzumab have also proved efficacy in MCF-7 models of *de novo* tamoxifen resistance and in BT-474 cells that overexpress HER2. The addition of either trastuzumab or lapatinib (a tyrosine kinase inhibitor which interrupts the HER2 growth receptor pathway) to aromatase inhibitor therapy has improved survival in patients with ER(+)/HER2(+) metastatic breast cancer [109]. Moreover, AEE788 (a combined inhibitor of EGFR, HER2 and VEGFR) plus tamoxifen or letrozole in breast cancer overexpressing HER2, may provide superior anti-tumour activity, compared with single agents [110].

As previously mentioned, the IGF1R pathway has a close bidirectional crosstalk with ER. Some studies have established that elevated IGF1R-promoted kinases contribute to the phosphorylation of ER. In turn, estrogens enhance expression of several IGF1R pathway components, like insulin-like growth factor II (IGF2), to reinforce IGF1R signaling [111]. The EGFR/IGF1R crosstalk in EGFR(+) tamoxifen-resistant variants of MCF-7 cells was studied. Although tamoxifen-resistant cells expressed reduced IGF1R protein levels compared with the wild-type MCF-7 cells, phosphorylated IGF1R protein levels were similar in the two cell lines under basal growth conditions, possibly as a consequence of increased IGF2 expression, which activated both IGF1R and EGFR. IGF2 promotes direct association of c-SRC with IGF1R, phosphorylated c-SRC, and increases EGFR phosphorylation at tyrosine 845, a c-SRC-dependent phosphorylation site [112]. A further mechanism observed in different tumor cell types is the ability of IGF2 to transactivate EGFR via metalloprotease-dependent release of the EGFR-ligands amphiregulin or HB-EGF [113]. ER(+) MCF7 cells with ectopic expression of IGF1R, are highly resistant to tamoxifen and fulvestrant, but in response to IGF1 ligand stimulation, displayed enhanced downstream activation of MAPK and PI3K signaling pathways, this was independent of ER signaling. Intriguingly, an agonistic behavior of tamoxifen at low doses was triggered in the presence of IGF1 [114]. In patients with tamoxifen-resistant tumors, the tumors with higher IGF1 ligand and ERα expression took longer to develop tamoxifen resistance, while tamoxifen-resistant tumors had lower IGF1 and ERα expression compared to tamoxifen-sensitive tumors [115]. However, while inhibitors of this pathway are currently in different trials, early reports have shown that IGF1R-specific inhibitors (like AG1024 and AEW541) or an IGF2 neutralizing antibody inhibited basal IGF-IR, c-SRC, AKT and EGFR phosphorylation, and significantly reduced tamoxifen-resistant basal cell growth. The c-SRC inhibitor SU6656 also inhibited growth, reduced basal and IGF2-induced c-SRC and EGFR phosphorylation, and blocked EGFR activation by TGFα. Similarly, AG1024 and SU6656 inhibited basal and IGF2-induced phosphorylation of c-SRC and EGFR, and SU6656 reduced TGFα-induced EGFR activity in tamoxifen-resistant T47D cells. Interestingly, AEW541 also inhibited insulin- and IGF2-stimulated effects in tamoxifen-resistant cells [112,116].

Recently, the estrogen receptor coactivator MED1 proved to be a novel crosstalk point for the HER2 and ERα pathways in breast cancer, showing a key role for MED1 in HER2-mediated tamoxifen resistance [117]. Indeed, MED1 expression positively correlated with HER2 status of the tumors, being highly phosphorylated in a HER2-dependent manner. Interestingly, downregulation of MED1 sensitized HER2-overexpressing cells to tamoxifen treatment, probably as a result of less MED1 recruitment to HER2 promoter, an event that is required for its expression. This is in accordance with previously mentioned gefitinib-dependent restoration of tamoxifen response in HER2-overexpressing tamoxifen-resistant MCF-7 cells.

#### 3.3.2. PI3K Cell Survival Pathways

ER activity is also associated to the PI3K pathway, which is activated by tyrosine kinase receptors in response to growth factors. The PI3K/AKT signaling pathway has been extensively investigated for its role in oncogenic transformation. It is very important in regulating proteins that control cellular proliferation such as cyclins, cyclin-dependent kinases and cyclin-dependent kinase inhibitors [118]. AKT is one of the downstream targets of PI3K, promotes cellular proliferation and anti-apoptotic responses [119]. Recent studies have indicated that estrogen stimulates association of ERα with IGF-1 receptor and p85 regulatory subunit of PI3K in the plasma membrane [120,121], which leads to AKT activation and its subsequent downstream effects. In turn, AKT phosphorylates nuclear ERα at serine 167, resulting in ligand-independent activation [52,122].

In various experimental models it has been demonstrated that PI3K pathway activation confers antiestrogen resistance. Knockdown of the suppressor phosphatase and tensin homolog (PTEN, a negative regulator of AKT), increased PI3K and AKT phosphorylation in ER(+) breast cancer cell lines, resulting in hormone-independent growth and resistance to tamoxifen and fulvestrant [123,124]. Endocrine-resistant tumor cells display upregulation of IGF-1R, HER2, and EGFR levels, as well as increased PI3K/AKT/mTOR activation. Clinical evidence suggests that activation of PI3K via either overexpression of HER2 or fibroblast growth factor receptor (FGFR)1, or loss of inositol polyphosphate-4-phosphatase type II (INPP4B), also confers antiestrogen resistance to patients with ER(+) breast cancer [125]. Interestingly, mutations in the PI3K alpha catalytic subunit (PI3KCA) is the most common genetic abnormality identified in ER(+) breast cancer (~30%), whereas PTEN loss is more associated with ER(−) disease. Such mutations in PI3KCA correlate with good long-term outcome in the patients. However, conflicting data exists in the literature regarding the prognostic implications of PI3KCA mutation in breast cancer and it will have to be further analyzed [126]. The addition of PI3K pathway inhibitors increases the pro-apoptotic effects of tamoxifen, primarily in the cell line with the highest endogenous levels of AKT activity, supporting the notion that either high expression of AKT or altered activity of the PI3K/AKT pathway could be associated with endocrine resistance [52,121]. Similar results in four different cell lines tested showed that the combination of BEZ235 (PI3K/mTOR inhibitor) and tamoxifen inhibited growth more than either tamoxifen or BEZ235 alone [127]. In another study, patients with ER(+) tumors were randomly assigned to neoadjuvant letrozole with or without everolimus inhibitor for four months before surgery. The combination therapy induced a higher clinical response and greater suppression of tumor cell proliferation compared with letrozole alone. Patients with advanced breast cancer who had progressed while receiving an AI were randomly assigned to tamoxifen with or without everolimus. They showed a significantly improved rate of clinical benefit, time to progression and disease-free survival with the combination, in comparison with tamoxifen alone [128–130].

Studies in human breast cancer cells subjected to long-term estrogen deprivation (LTED) have demonstrated that these cells first develop hypersensitivity to low-dose estrogens and then become estrogen independent. This phenomenon seems to contribute to resistance to endocrine therapy, which may result in part by increased levels of ER and upregulation of the MAPK, PI-3-kinase and mTOR growth factor pathways and phosphorylation of ERα [131–136]. Studies in MCF-7 cells treated long-term with tamoxifen showed increased MAPK and aromatase activity during the acquisition of tamoxifen resistance. This may occur either as response to estrogen deprivation or interruption of the process of estrogen signaling [135,137]. In fact, three stages have been suggested that lead to tamoxifen resistance in cells with long-term exposure to tamoxifen: in stage 1, tamoxifen behaves as an estrogen antagonist; in stage 2, the tumors increasingly become sensitive to the agonistic effects of tamoxifen; and, in stage 3, the tumor has increased sensitivity to estrogen (hypersensitivity).

The PI3K/mTOR inhibitor BEZ235 and the TORC1 inhibitor everolimus (mTOR inhibitor, also known as RAD001) inhibit growth of LTED cell lines [138]. A recent study in ER(+) breast cancer cell lines before and after LTED or short-term estrogen deprivation (STED) showed that the treatment with the PI3K catalytic subunit inhibitor BKM120, the RAD001 inhibitor and the dual PI3K/mTOR inhibitor BGT226 induced apoptosis. Additionally, fulvestrant alone did not promote apoptosis in STED cells or LTED cells. However, when fulvestrant was used in combination with BGT226, BKM120 and RAD001, it potentiated apoptosis of these inhibitors in MCF7 LTED cells. In contrast, treatment with fulvestrant did not promote apoptosis in the ER-negative T47D LTED cells with any of the three agents tested. These data suggest that fulvestrant may sensitize cells to the therapeutic effects of PI3K inhibitors under circumstances where resistance to estrogen deprivation is associated with ligand-independent ER activity [139]. The crosstalk between ER and PI3K/AKT pathways shows a new strategy in the combat against endocrine resistance.

#### 3.3.3. Stress-Activated Protein Kinase/c-junNH2 Terminal Kinase Pathway

Stress-activated protein kinase/c-junNH2 terminal kinase pathway may interact with ER either by binding with the AP-1 transcription complex or by direct p38 MAPK activation. AP-1 is a complex of proteins composed of Jun and Fos family members of nuclear phosphoproteins, which dimerize and bind to DNA consensus sequences TGAC/GTCA (AP-1 sites) and modulates expression of the target genes [140]. AP-1 transcriptional activity is increased either by their phosphorylation through the JUN NH2-terminal kinases (JNKs) or stress-activated protein kinases (SAPKs) or by increased abundance of any of its components [141]. One study reported that the development of tamoxifen resistance in MCF-7 cells is accompanied by enhanced AP-1 DNA binding [142]. It was also observed in a panel of 30 ER(+) primary human breast tumors with acquired tamoxifen resistance, as compared to a matched panel of 27 untreated control tumors [143]. Similarly, a study in a xenograft mouse model showed that tamoxifen-resistant tumors increased AP-1 dependent transcription and phosphorylated c-Jun and JNK levels, compared with estrogen treated tumors. Apparently, the tamoxifen resistant phenotype is associated with enhanced oxidative stress, leading to activation of JNK and increased AP-1 activity by an agonistic effect of tamoxifen at AP-1 sites [144–146].

On the other hand, activation of the p38 MAPK pathway has been reported to occur in response to environmental stress including ionizing radiation, heat, oxidative stress, inflammatory cytokines, growth factors, and tissue ischemia [147,148]. In clinical breast cancer specimens and in xenograft tumors resistant to tamoxifen high levels of p38 MAPK expression have been found [72]. The increase in p38 MAPK levels produced by tamoxifen, may account for the partial agonist activity of this drug. In endometrial cancer cells, p38 can phosphorylate and activate ER, inhibit ER nuclear export, enhance interaction of ER with coactivators, and increase the agonistic activity of tamoxifen [49,149], perhaps these mechanisms may well be important for tamoxifen resistance in clinical breast cancer. Moreover, inhibition of p38 by SB202190 reduces the proliferation of breast cancer cell lines. Studies *in vitro* and *in vivo* have shown that pharmacological inhibition of p38 by RWJ67657 (other p38 inhibitor) resulted in inhibition of the downstream p38 targets hsp27 and MAPK. Furthermore, decreased biological effects of p38, including ER-mediated gene expression and clonogenic survival in a dose-dependent manner were also observed [150]. Then, targeting this signaling pathway may be beneficial to overcome tamoxifen resistance [151].

#### 3.3.4. Other Signal Transduction Pathways

Other signal transduction pathways related to the endocrine resistance are nuclear factor-κB (NFκB), Notch, keratinocyte growth factor (KGF), and platelet-derived growth factor (PDGF)/Abl signaling pathway [152–154].

NFkB stimulates cell proliferation and invasion by induced genes such as cyclin D1 and urokinase-type plasminogen activator (uPA). NFkB activation has been associated with progression of hormone-independent breast cancers. Low NFkB activation was found in ER(+) breast cancers cells but constitutively elevated in ER(−) breast cancers cells [155,156]. Moreover, in ER(+) breast cancers has been demonstrated that tamoxifen can activate NFκB, stimulate cell growth and survival, and thereby contribute to endocrine resistance [157]. Crosstalk between ER and NFκB pathways that contributes to the development of endocrine resistance was reviewed in Sas *et al.* [158].

KGF pathway can stimulate the growth of mammary epithelium by inducing aromatase activity, thereby promoting conversion of androgens to estrogens in primary cultured human breast cells. KGF might increase the endocrine resistance via decreasing ER, progesterone receptor (PR) and protein tyrosine phosphatases gamma (PTPγ). The presence of ER and PR is a good prognostic factor and indicator of benefit from endocrine therapy. Low levels of ER and PR in human breast cancers have been associated with resistance to tamoxifen and increased risk of breast cancer. On the other hand, PTPγ has been proposed as a candidate tumor suppressor gene in kidney, ovarian and lung tumors and may play an important role in neoplastic processes of human breast. This pathway suggests novel strategies for breast cancer treatment via interference with KGF signaling [159].

It has also been found that the Notch pathway is implicated in both cell fate in the normal human mammary gland and regulation of cancer stem cells (CCs) in both ductal carcinoma *in situ* and invasive carcinoma of the breast [152,160,161]. Binding of Notch ligand (Jagged or Delta) to the Notch receptor cleaves its intracellular domain (NICD), which translocates to the nucleus where it binds with co-activators to induce transcription of its target genes and regulates migration and invasion of breast cancer cells. Estradiol inhibits Notch activity, and affects Notch receptor cellular distribution. Tamoxifen and raloxifene block this effect, reactivating the Notch pathway. Pharmacologic inhibition of Notch activation with γ-secretase inhibitors was more effective in combination with tamoxifen that tamoxifen alone [162]. These data indicate that γ-secretase inhibitors block the proliferative effect of tamoxifen through Notch pathway, and at the same time allow tamoxifen to exert its antagonistic effect.

PDGF/Abl canonical signaling pathway has been also associated to resistance [153]. PDGF receptor (PDGFR) is classified as a tyrosine kinase receptor whose activation is dependent on the binding of PDGF resulting in stimulation of several intracellular pathways. PDGF can promote tumor growth via autocrine stimulation of malignant cells, overexpression or overactivation of PDGFRs, or by stimulating tumor angiogenesis. Abl is a Src-like nonreceptor protein kinase that acts downstream of the PDGFR [163]. Abl is involved in the regulation of cell proliferation, apoptosis, adhesion, cell migration and stress response [164,165]. *In vitro* and *in vivo* studies have demonstrated a novel interaction between ER and the PDGF/Abl signal transduction pathway during adaptation to LTED and which appears partly responsible for the resistant phenotype. These data suggest that this pathway may provide a novel biomarker of early resistance to AI therapy [153]. Despite the success of AIs in treating ER(+) breast cancer, approximately 15%–20% of patients that receive this treatment relapse within 5–10 years of therapy initiation because of either selection of cells insensitive to estrogen or activation of signaling by non-endocrine pathways [132,166].

### 3.4. Altered Expression of Specific microRNAs

MicroRNAs (miRs) are small regulatory and non-coding RNA molecules of 19–24 nucleotides in length that modulate the expression level of specific proteins based on sequence complementarities with their target mRNA molecules. This process occurs by either degrading the target messenger RNA (mRNA) or suppressing the protein synthesis [167]. miRs have been shown to regulate a variety of cellular processes such as differentiation, cell growth, and cell death [168]. miR genes are frequently located at cancer-associated genomic regions and the deregulation of its expression has been found to contribute in the tumorigenic process. Recent research suggests miRs as cancer prognostic biomarkers and therapeutic targets [169–171].

Altered expression of specific miRs has been implicated in tamoxifen resistance development and predicts outcome and therapeutic response in breast cancer [172–174]. Different miR expression profiles have been identified in tamoxifen resistant and sensitive breast cancer cell lines by microarray analysis (Table 1). By using this technique, 97 miRs differentially expressed in MCF-7 endocrine-sensitive *versus* resistant LY2 breast cancer cells were identified. Opposite expression of miR-10a, miR-21, miR-22, miR-29a, miR-93, miR-125b, miR-181, miR-200a, miR-200b, miR-200c, miR-205, and miR-222 in MCF-7 *versus* LY2 cells was confirmed by quantitative real-time PCR [175].

As previously mentioned, increased growth factor signaling (in particular the EGFR/HER2 pathway), the estrogen agonist activity of tamoxifen in breast cancer cells that express high levels of HER2, aberrant expression cell cycle regulators and lack of expression of ERα in breast cancer are factors that contributes to tamoxifen resistance [97,180–182]. In this regard, altered expression of miR-221 and miR-222 (miR-221/222) has an important role. The miR-221/222 expression levels were significantly elevated in HER2(+) breast cancer cells compared with cells with low or negative HER2 expression. Ectopic expression of miR-221/222 in tamoxifen sensitive cells resulted in downregulation of the cell cycle inhibitor p27^kip1^, and conferred resistance to tamoxifen [176]. p27^kip1^ is a target of miR-221/222 and its overexpression induced enhanced cytotoxicity in tamoxifen resistant breast tumor cells [176]. Also, aberrant expression of miR-221/222 has been associated with fulvestrant resistance by activating β-catenin and modulating TGF-β and p53 signaling [183]. In addition, miR-221/222 are highly expressed in ERα negative breast cancer cells. Knockdown of these two miRNAs partially restored ERα protein expression and tamoxifen induced cell growth arrest and apoptosis. In contrast, overexpression of miR-221/222 in ERα(+) cells reduced levels of this receptor and caused resistance to tamoxifen [184]. Indeed, several miRNAs were reported to be associated with the regulation of ERα expression [185–188]. These results indicate that miR-221/222 play an important role in the regulation of signaling cascades, control of cell proliferation and, regulation of ERα expression; which altogether impact anti-estrogen resistance.

HER2Δ16 is the oncogenic splice isoform of HER2, commonly coexpressed with HER2 in ER(+) breast tumors; its expression promotes estrogen-independent growth and tamoxifen resistance [189,190]. The mechanism of endocrine-resistance in HER2Δ16-expressing cells involves the upregulation of anti-apoptotic BCL-2 protein induced by tamoxifen and suppression of miR-15a and miR-16 (miR-15a/16) levels. Overexpression of miR-15a/16 reduced tamoxifen-induced BCL-2 expression and sensitized HER2Δ16-expressing cells to tamoxifen response. Inversely, miR-15a/16 inhibition in tamoxifen-sensitive cells increased BCL-2 expression and promoted tamoxifen resistance [190].

Altered expression of miR-342 has been identified in tamoxifen-resistant breast tumor cells. miRNA expression profiles of tamoxifen sensitive- and resistant-MCF-7 variants, including those expressing HER2Δ16, showed significant suppressed levels of miR-342 in tamoxifen-resistant cells. Moreover, miR-342 reduced expression was associated with tamoxifen failure in primary human breast tumors and downregulation of miR-342 in tamoxifen sensitive cells conferred tamoxifen resistance. In contrast, overexpression of miR-342 sensitized resistant breast tumor cells to tamoxifen induced apoptosis and inhibition of cell proliferation [177].

14-3-3ζ acts as a suppressor of apoptosis and has an important role in tumorgenesis in multiple types of cancer. Its overexpression has been found in 42% of breast tumors [191]. Previous studies demonstrated that 14-3-3ζ gene expression was upregulated by tamoxifen in ER(+) breast cancer cells and it was associated with poor outcome on endocrine therapy [192]. The mechanism by which tamoxifen regulated 14-3-3ζ levels, involves the downregulation of miR-451 that specifically targets 14-3-3ζ. The regulation of 14-3-3ζ and miR-451 was selective for tamoxifene because neither raloxifene nor fulvestrant had an effect on miR-451. Overexpression of miR-451 and downregulation of 14-3-3ζ expression in endocrine-resistant cells restored the effectiveness of tamoxifen to decrease cell proliferation, increase apoptosis and reduce activation of EGFR/HER2 signaling. The loss of miR-451 and upregulation of 14-3-3ζ induced by tamoxifen seems to be an additional mechanism by which ER(+) breast cancer cells develop resistance to tamoxifen therapy [179,193].

miR-210 is involved in different biological processes such as cell proliferation, migration and invasion, High miR-210 expression was associated with higher risk of recurrence in ER(+), tamoxifen treated breast cancer patients [194]. Other miRs related to therapeutic response are miR-301 and miR-101, which overexpression was associated with tumor growth and resistance to tamoxifen [195,196]. In MCF-7 cells, miR-101 promoted estrogen-independent growth and caused upregulation of phosphorylated Akt. MiR-101 activated Akt through targeting membrane-associated guanylate kinase (MAGI-2), an essential protein in the activity of the tumor suppressor PTEN. Upregulated miR-101 suppressed PTEN activity and facilitated Akt activation, which promoted the estrogen independent growth of breast cancer [196]. Also, miR-301 overexpression was associated with resistance to tamoxifen and breast cancer progression. miR-301 acts through multiple pathways and oncogenic targets, including PTEN, the transcription factor FoxF2, the pro-apoptotic protein BBC3, and the collagen, type II, alpha 1 (CoL2A1). Furthermore, miR-301 works in cooperation with SKA2 (essential for proper chromosomal segregation) contributing to breast cancer progression [195].

Some evidence has suggested that the effect of antiestrogens in breast cancer is mediated in part by regulation of TGF-β signaling pathway [197–200]. Yoo *et al.* demonstrated that the enhanced activation of Akt results in loss of TGF-β response induced by tamoxifen, and this is associated with the development of tamoxifen resistance in breast cancer [201]. In contrast, overexpression of TGF-β has been reported to mediate tamoxifen resistance *in vivo* by abrogating natural killer (NK) cell function involved in the antitumor effect of tamoxifen [202]. Human miR-128a has been associated with breast cancer aggressiveness [203] and was predicted to target the TGF-β signaling pathways members (TGF-β receptor I (TGFβRI) and the transcription factor SMAD2) [204]. Particularly, miR-128a targets the 3′UTR region of the TGFβRI and negatively regulates TGFβRI protein expression. The miR-128a expression levels were found significantly elevated in letrozole-resistant breast cancer cells compared with other resistant cell lines, suggesting functional specificity of this miR in letrozole-resistance. Moreover, downregulation of endogenous miR-128a resulted in re-sensitization of the aromatase inhibitor-resistant breast cancer cell line to the growth inhibitory effect of TGFβ [204].

### 3.5. Coregulatory Proteins

Tamoxifen acts as an ER antagonist in breast cancer but as an agonist in other tissues such as uterus, cardiovascular system, and bone. These differences in tamoxifen activity could be explained by several mechanisms [205]. One of these mechanisms involves changes in the level of expression of coregulatory proteins (coactivators and corepressors) that can influence regulation of ER transcriptional activity [206,207].

The ER coactivator AIB1 (also known as SRC-3) is considered to be a proto-oncogene, which is overexpressed in more than 30%, and genetically amplified in 5%–10%, of breast tumors [208–211]. High levels of ER coactivators may enhance the estrogen-agonist activity of tamoxifen and contribute to tamoxifen resistance [31,206,212]. High SRC-3 expression has been associated with poor overall breast cancer patient survival [213]. Moreover, overexpression of SRC-3 along with HER2 converts tamoxifen into an agonist with increase in the molecular crosstalk between the ER and HER2 pathway [97]. Further, it has been shown that tamoxifen induces ERα-SRC-3 interaction in HER2(+) human breast cancer [214]. In contrast, dissociation of SRC-3 from ER has been shown to restore sensitivity in tamoxifen resistant cells [215]. In a clinical study, patients who had received adjuvant tamoxifen with tumor overexpressing SRC-3 along with HER2 displayed worse disease-free survival. Better prognosis and longer disease-free survival was observed in patients not receiving adjuvant tamoxifen therapy whose tumors expressed high levels SRC-3 [101]. Other study confirmed that high SRC3 expression was a marker of tamoxifen resistance in HER2 overexpressing. Moreover, patients who had received adjuvant tamoxifen with high SRC-3 expression along with one or more of EGFR- or HER3-overexpressing tumors was associated with an increased risk of relapse [216]. In surgical specimens from ER(+) breast cancer patients who received neoadjuvant tamoxifen therapy, tamoxifen significantly increased the expression of SRC-1, SRC-2 and SRC-3 [217]. SRC-1 and SRC-3 protein levels were higher in an endocrine resistant cell lines in comparison with endocrine sensitive cells. Knockdown of SRC-1 and SRC-3 resensitized endocrine resistant cells to tamoxifen treatment. Also, colocalization of SRC-1 and SRC-3 with ERα was increased in endocrine resistant cells following treatment with tamoxifen in comparison with endocrine sensitive cells. These data implicate the involvement of inappropriate interactions between ER and its coactivator in the endocrine resistance [218]. Recent results showed that the expression SRCs, HER2 and HER3 is stimulated by tamoxifen treatment in DMBA induced breast cancer [219]. Knockdown of SRC in endocrine-resistant, HER2(+) cells restored the effectiveness of tamoxifen to decrease cell proliferation [214].

HER2 could be repressed by both estrogen and tamoxifen, thus the anti-proliferative effects of tamoxifen could be in part due to repression of HER2 [220–222]. Paired Box 2 (Pax2) was found to be an important mediator of repression of HER2 in ER-positive breast cancer cells. After tamoxifen treatment, Pax2 is recruited to the ER functioning as an ER-associated transcriptional repressor. Knockdown of Pax2 resulted in elevated HER2 transcription and protein levels in the presence of both estrogen and tamoxifen and reversed the growth inhibitory effects of tamoxifen. Overexpression of SRC-3 blocked Pax2 binding to ER, resulting in increased HER2 transcription and cell proliferation in the presence of tamoxifen. Furthermore, tamoxifen resistant cells with elevated HER2 levels showed low Pax2 protein levels and tamoxifen treatment increased SRC-3 recruitment. In contrast, when overexpressing Pax2 in these cells, tamoxifen decreased SRC-3 binding to the ER and repressed HER2 mRNA and protein levels, the growth inhibitory effects of tamoxifen were also restored. This suggests that SRC-3 competes with Pax2 for binding and regulating the HER2 gene. [222]. Interestingly, patients receiving tamoxifen with ER positive tumors with Pax2 positive and SRC-3 negative had the best prognosis compared with patients with Pax2 positive and SRC-3 positive tumors or patients with Pax2 negative tumors. The lowest percentage of HER2 positivity was observed in patients with Pax2 positive and SRC-3 negative tumors [222]. These data confirm an essential role for SRC-3 and HER2 in tamoxifen resistance in ER positive breast cancer cells.

Increased SRC-1-ERα interactions are observed in patients who are resistant to treatment [218]. As the tumor progresses, however, increasing evidence suggests that SRC-1 engages in transcriptional interactions independently of ER. A new ER-independent target of SRC-1, the ADAM22 protein involved in cell adhesion, spreading and migration, was identified [223,224]. Evidence from ChIP-seq, bioinformatics and molecular studies suggested that SRC-1 can regulate ADAM22 independently of ER in endocrine-resistant cells [223].

The role of coactivators in resistance to AI therapy has also been documented. Flageng *et al.* demonstrated that co-activators and HER2 are upregulated in the tumors from breast cancer patients during neo-adjuvant treatment with aromatase inhibitors [225]. Moreover, expression of AIB1 was associated with disease recurrence and reduced disease-free survival time in patients treated with an AI as first-line therapy [226]. In AI resistant cells, increased cell migration and loss of differentiation were found in comparison with endocrine sensitive cells. SRC-1 was an important modulator of this aggressive phenotype. AI treatment interacts with MAPK-activated Ets2 transcription factor regulating expression of Myc and MMP9 [227].

The nuclear receptor corepressor (NCoR) has been considered as an important predictor of the tamoxifen response [228,229]. In a xenograft model for human breast cancer, NCoR levels decreased in the tumors that acquired resistance to tamoxifen [230]. In a clinical study, patients with the best prognosis had high NCoR expression levels and normal HER2 expression [231]. Another study in ERα(+) breast tumors from postmenopausal patients treated with tamoxifen after surgery demonstrated that low NCoR expression was associated with significantly shorter relapse-free survival [229]. Higher NCoR expression levels in tumors from patients without recurrence compared with patients with recurrence supporting its role as a tamoxifen resistance mechanism [232].

The Tab2 protein is a facultative component of the NCoR complex [233] and was found as an important mediator of resistance to endocrine therapy [234]. Interaction between Tab2 and ERα was observed in tamoxifen resistance cells and interfering with this binding restored tamoxifen response. Furthermore, downregulation of Tab2 increased the antiproliferative response to tamoxifen [234]. Tab2 interacts with ERα/NCoR and causes dismissal of NCoR, leading to loss of the antiproliferative response in prostate cancer cells [235]. This issue has not yet been addressed in breast cancer cells and deserves further investigation.

Tamoxifen-sensitive cells showed high NCoR binding to the ER in comparison with tamoxifen-resistant cells. Combination treatment with either tamoxifen and IKK inhibitor parthenoide (PA), or tamoxifen and proteasome inhibitor bortezomib (PS341), which were capable of suppressing NFkB and AP-1, regulates gene expression. These combinations were able to restore the levels of NCoR binding to tamoxifen-liganded ER in the tamoxifen-resistant cells [154].

Taken together, these data raise the possibility that progressive reductions in corepressor activity during tamoxifen therapy may enhance the agonist effects of tamoxifen on the ER contributing to tamoxifen resistance.

### 3.6. Tamoxifen Metabolism and Genetically Based Resistance

The body converts drugs to metabolites that are more water soluble and thus more easily excreted. Nevertheless, during phase I reactions, some drugs may turn either into toxic metabolites or into more active compounds with significant therapeutic benefit [236]. Tamoxifen is metabolized in the liver mainly by two P450 cytochromes: CYP3A4 and CYP2D6, which produce *N*-desmethyltamoxifen and 4-hydroxytamoxifen, respectively. Afterwards, further oxidation of these metabolites results in the formation of a very active metabolite: 4-hydroxy-*N*-desmethyltamoxifen, also known as endoxifen, which production is mostly catalyzed by CYP2D6 [237,238]. Endoxifen and 4-hydroxytamoxifen have greater binding affinities for ERs and suppress cell proliferation more effectively than tamoxifen itself. Nevertheless, endoxifen plasma concentrations are 5–10-fold higher than those of 4-hydroxytamoxifen and are about 100 times more potent than tamoxifen in anti-estrogen activity [239,240]. Therefore, endoxifen is thought to be the responsible metabolite for the pharmacological activity of tamoxifen *in vivo*. Genetic variants of the cytochromes involved in tamoxifen metabolism may affect the final therapeutic response. Indeed, polymorphism in the gene coding for CYP2D6 significantly affects enzymatic activity, providing a potential mechanism for drug resistance. More than 75 CYP2D6 alleles have now been described and classified depending on their effects on CYP2D6 enzyme activity [241]. In this manner, extensive, intermediate or poor metabolizer alleles have been defined depending on how effectively tamoxifen is metabolized, which finally results in differential therapeutic responses to standard doses of the drug [236,241]. Therefore, CYP2D6 genotyping is advised in order to guide the correct selection and dosing of tamoxifen in a tailored manner. In this respect, a tool to simplify genotype interpretation has been designed and is known as “CYP2D6 activity score system” [242], which is useful to personalize drug therapy. Indeed, the use of pharmacogenomics in oncology is paramount to predict drug response or toxicity [243].

On the other hand, individual variations in the activity of phase II enzymes may partially explain final endoxifen bioavailability. For instance, women with high activity of UDP-glucuronosyltransferase-2B15 (UGT2B15) genotype had a worse recurrence-free survival than those with wild type alleles [244,245]. Inasmuch, an association between the rs9282861 homozygous variant AA genotype of sulfotransferase 1A1 (SULT1A1, involved in detoxification of 4-hydroxytamoxifen), and overall long-term survival of breast cancer patients treated with adjuvant tamoxifen, has been described [246]. Therefore, these findings suggest that polymorphism in the genes coding for phase II enzymes alter the long-term clinical outcome of patients receiving tamoxifen, and might explain some cases of resistance to therapy.

While tamoxifen is mainly metabolized in the liver, tumor cells may also contribute to this process by expressing functional P450 species. Indeed, the impaired ability of a tumor to efficiently bioactivate tamoxifen is another mechanism to explain resistance to therapy. In this respect, gene therapy holds promise as a new approach to achieve differential P450 cytochromes expression in order to bypass drug resistance [247,248]. The main purpose of this strategy is to selectively increase tumor cell exposure to the active metabolites generated locally by an exogenous P450 gene. This approach has been previously tested with cyclophosphamide [249], which is a prodrug bioactivated by P450 enzymes, as in the case of tamoxifen. Moreover, the efficacy of gene therapy can be enhanced by further increasing intra-tumor metabolism by means of co-expressing the P450 cytochrome together with the flavoenzyme NADPH-P450 reductase, which increases P450 metabolic activity [247]. Prominent among the benefits of this strategy are: enhanced therapeutic effect without increasing host toxicity and the possibility to use well-established and clinically effective anticancer prodrugs [248]. It is noteworthy to mention that even if localized CYP3A4 and CYP2D6 overexpression and activity might benefit patients under tamoxifen treatment, other anti-neoplastic drugs might easily be inactivated by these cytochromes, limiting the success of combined chemotherapy. For instance, low CYP3A4 expression in breast tumors resulted in a better response to docetaxel [250,251], while high CYP3A expression in osteosarcoma tumors predicted metastasis and poor prognosis [252]. This is explained by the fact that CYP3A4 catalyzes the oxidation of drugs frequently used as chemotherapeutic agents, such as doxorubicin and many others, whereas tamoxifen, as a prodrug, needs to be activated to achieve therapeutic results.

Another factor that plays a role in the response of patients to drug treatment is the concomitant administration of other drugs. Indeed, medications prescribed to patients on tamoxifen therapy can also block endoxifen production by inhibiting CYP2D6, as in the case of some antidepressants [240]. Indeed, selective serotonin and noradrenaline reuptake inhibitors are potent CYP2D6 inhibitors. For instance, fluoxetine and paroxetine may confer a poor metabolizer phenotype. This is important since these drugs are usually prescribed to patients under tamoxifen treatment, in order to alleviate some of its undesirable effects such as hot flashes [240], but might also be the cause of endocrine resistance, and, therefore, its concomitant prescription with tamoxifen should be carefully evaluated.

In summation, the probability of genetically based resistance to tamoxifen should be taken into consideration when designating a therapeutic approach. Although multiple mechanisms might be involved in hormonal resistance in patients, an interesting approach to overcome is the combined therapy, which is discussed next.

## 4. Combination Therapy

Combination regimens significantly improve the clinical outcome—especially in metastatic breast cancer. Therefore, drug combination regimens appear to be a good approach to delay cancer adaptation to treatment and secondary resistance. A common strategy designed to clinically address *de novo* and acquired endocrine therapy resistance is the use of combined hormonal treatment with targeted anti-HER2, anti-EGFR or multikinase inhibitor therapy [253]. Regarding hormonal treatment, several factors are being used in order to interfere with the estrogen signaling pathway, specifically: pure ER antagonists such as fulvestrant, and SERMs such as tamoxifen. Additionally, estrogen production might be blocked by using aromatase inhibitors (AI) such as anastrozole, exemestane and letrozole [254]. On the other hand, drugs that interfere with growth factor signalization include trastuzumab, gefitinib, lapatinib, inhibitors of farnesyltrasferase, inhibitors of the mammalian target of rapamycin and multitargeted tyrosine inhibitors [253]. Endocrine therapy is also being clinically tested in combination with antiangiogenic factors such as bevacizumab (a humanized monoclonal antibody that inhibits VEGF-A) and motesanib (an oral inhibitor of VEGF receptors) [253,255,256].

In general, interfering with both estrogen and growth factor signaling pathways by dual pharmacological inhibition seems to be more effective than monotherapy to inhibit tumor growth, especially for ER(+)/HER2(+) tumors. For example, combination of trastuzumab plus letrozole provides superior benefit over each drug alone in a MCF-7 xenograft model once acquisition of resistance is established by increased HER2 expression [257]. Moreover, lapatinib, an oral dual selective inhibitor of the tyrosine kinase domain of EGFR and HER2, increased the sensitivity of breast cancer cells to 4-hydroxy-tamoxifen, restoring endocrine sensitivity [258]. Similarly, synergistic effects of lapatinib in combination with fulvestrant have been reported in breast cancer cell lines expressing differential amounts of ER, HER2 and EGFR [259]. Clinically, the combined treatment with HER2 blockade and hormonal/AI therapy offers clinical advantages beyond those provided by endocrine or AI therapy alone in ER(+)/HER2(+) breast tumors. In these particular cases, it is known that gefitinib may eliminate the crosstalk between HER2 and ER signaling cascades. Indeed, tamoxifen behaves as an estrogen agonist in breast cancer cells that express high levels of HER2, resulting in *de novo* resistance. Gefitinib treatment reestablishes corepressor complexes with tamoxifen-bound ER on target gene promoters, eliminating tamoxifen agonistic effects, and restoring its antitumor activity both *in vitro* and *in vivo* [97]. The combination of trastuzumab plus an AI significantly improved progression-free survival, response rates, and clinical benefits when compared with AI monotherapy in postmenopausal women [260]. This might be explained by the fact that unlike tamoxifen, AIs have no ER agonistic activity, since their mechanism of action consists in blocking the final enzymatic step in the biosynthesis of estrogens and thus inhibit estrogen production.

## 5. Conclusions

The resistance to endocrine therapy involves multiple mechanisms including, at least in part, a switch from steroid signaling to growth factor signaling leading to steroid-independent tumors. Because breast cancer is a highly heterogeneous pathology both at the clinical and molecular level, the pharmacological prescription must be as tailored as possible. Therefore, the molecular signature of each tumor should be determined in order to accurately select the therapeutic approach, predict prognosis and response to therapies. The combination of endocrine therapy with other drugs targeting key molecules involved in hormone resistance is the most promising approach to prevent and/or overcome endocrine resistance and benefit these breast cancer patients.

## Figures and Tables

**Table 1:** miRs expression profiles in tamoxifen-resistant breast cancer cells. miR overexpression (at least >1.5 fold higher), and underexpression (50% lower), in comparison with tamoxifen-sensitive breast cancer cells.

Overexpression	Underexpression	Reference
miR-221	miR-342	[176,177]
miR-222	miR-489	[176]
miR-181	miR-21	[176,178]
miR-375	miR-24	[176]
miR-171	miR-27	[176]
miR-213	miR-23	[176,177]
miR-203	miR-200	[176]
miR-321	miR-451	[176,179]
miR-1308	miR-1180	[177]
